# Fasting Ramadan During COVID-19 Pandemic: Immunomodulatory Effect

**DOI:** 10.3389/fnut.2020.557025

**Published:** 2020-11-05

**Authors:** Taghreed Abunada, Hanan Abunada, Hatem Zayed

**Affiliations:** ^1^Biomedical Science Department, College of Health Sciences, QU-Health, Qatar University, Doha, Qatar; ^2^Qatar Biomedical Research Institute, Hamad Bin Khalifa University, Doha, Qatar

**Keywords:** fasting Ramadan, COVID-19, immune, diet, restriction

## Abstract

As of April 24, 2020, more than 1. 6 billion Muslims observe the holy fasting month of Ramadan worldwide. The safety of fasting of healthy adult Muslims during the COVID-19 era is debatable. In this perspective, we discuss the available scientific evidence of the advantages of fasting against COVID-19.

## Fasting Ramadan During COVID-19 Pandemic

As of April 24, more than 1.6 billion Muslims are celebrating the fasting month of Ramadan worldwide, where healthy adult Muslims practice diurnal intermittent fasting and refrain from eating and drinking from dawn to sunset (1). The safety of fasting of healthy Muslims is debatable as some argue that fasting will render them vulnerable to the severe consequences of COVID-19 (2–4), especially after the WHO recommendations for adult people to get a balanced diet and drink plenty of water throughout the day as protective measures against COVID-19 (5). Although following such measures would not eliminate the risk rather than improve the symptoms in case of infection, the recommendations might be misinterpreted to encourage some Muslims to stop fasting during Ramadan. Ramadan fasting is only required of healthy adult Muslims who are physically able to fast (6, 7). Therefore, Muslims are legally exempted from fasting if their health status would not enable them to fast. On the contrary, they are then required not to perform Ramadan fasting (2).

There are many patterns of intermittent fasting practiced worldwide, such as complete alternate day fasting, modified fasting regimens, and time-restricted fasting (8). Apart from the diurnal intermittent fasting of Muslims during the month of Ramadan, fasting is practiced by Christians, Jews, and other people worldwide for religious, spiritual, and health purposes (8).

## Fasting Immunomodulatory Effect in Experimental Studies

Alternate day fasting is an intermittent fasting approach that shows benefits on strengthening the immune system, alters the gut microbiota, and enhances antioxidative microbial metabolic pathways (9, 10). Alternate fasting–refeeding of BALB/c mice maintains the immune dynamics and hemostasis of B cells (11) and induces the migration of naïve B cells to the bone marrow, ready for combat (11–13). Alternate fasting–refeeding nutritional signals mediate this process and regulate it by CXCL13 expression (11). The CXCL13 chemokine reflects the germinal center activity where high-affinity antibody maturation and interaction with Th-cells occur (10). Diet restriction by 30% and 3 days of water-only fasting in C57Bl/6 mice increases the number of recirculating mature B cells in the bone marrow (14). A study by Nagai et al. examined the effect of alternate fasting/re-feeding for 24–36 h on BALB/c mice gut and found that such type of fasting decreases the number of Peyer's patches lymphocytes, while refeeding selectively restored naïve—but not germinal center and IgA class switched—B cells. The germinal center B cells underwent massive apoptosis during fasting due to downregulation of mTORC1 signaling, whereas naïve B cells shuttle between Peyer's patches and the bone marrow during these fasting–refeeding cycles in a CXCL-13-dependent manner (11). Calorie restriction and short-term fasting with access to water only for 48 h induce the cell cycle arrest of immature hematopoietic stem cells (HSCs) and increase the number of naïve CD8+/CD4+ T cells in the bone marrow of C57Bl/6 mice (15). Diet restriction by 30% and 3 days of water-only fasting increase mature CD3+ T cells in the bone marrow of C57Bl/6 mice (14). Diet restriction by 50% of C57BL/6NTac mice triggers memory T cell homing to the bone marrow and enhances their rapid protective function thereafter (16). Free access to water with food deprivation for 1 or 3 days augments the cytolytic activity of TRAIL-natural killer cells against virus-infected cells in C57BL/6J mice (17). Another study by Contreras *et al*. investigated the effect of calorie restriction in both mice and human and found that, during fasting, the T-lymphocytes redistribute and, upon re-feeding, undergo homeostatic proliferation, presumably to fill the void left by the redistributing cells in response to homeostatic cytokine, IL-7 (18). Cheng et al. examined the effect of prolonged fasting on murine HSCs and showed that prolonged fasting induced self-renewal and lineage-balanced regeneration of long-term HSCs and niche cells. This effect was mediated by many signal transduction changes of IGF1 and PKP activity (19, 20). Thus, these studies suggest that different fasting patterns preserve and improve the immunity by immune cell rejuvenation. Supplementary Table 1 describes the immunomodulatory effect of different fasting patterns in experimental studies.

Actually, intermittent fasting exerts well-being effect on different body organs and leads to a better metabolic health status in humans (21). Apart from its well-documented beneficial effects on the brain, heart, liver, intestine, and muscles, recent studies show that it ameliorates the immune system by rejuvenation and regeneration of stem cells, reducing inflammation and oxidative stress, reducing autoimmunity, improving cell repair (autophagy), and promoting healthy aging (21). These benefits encourage many physicians to recommend it as an alternative treatment for patients with asthma, hypertension, diabetes, rheumatoid arthritis, multiple sclerosis, systemic lupus erythematosus, and even cancer (21, 22).

Of interest is that diurnal intermittent fasting and its model of Ramadan affect the immunity by changing the body's response toward infection, inflammation, and oxidative stress (23). For instance, Ramadan fasting reduces the pathogenicity of *Mycobacterium tuberculosis* by increasing the macrophage number and INF-γ secretion in fasting volunteers (23). It was demonstrated to be safe for patients infected with HIV and on antiretroviral therapy (23). It decreases C-reactive protein, pro-inflammatory cytokines such as IL-1β, TNF-α, IL-6, and IL-8 (24–27), and pro-inflamatory CXC chemokines such as CXCL1, CXCL10, and CXCL12 (27). It has beneficial effects on nitric oxide and glutathione levels in women with polycystic ovary syndrome (28). It has a potential protective role against oxidative stress and inflammation by increasing the expression of three antioxidant genes (TFAM, SOD2, and Nrf2) (29). A systemic review by Adawi et al. illustrated a graphical explanation of the mechanism related to Ramadan intermittent fasting and the immune system (6).

## Fasting Potential Favorable Effect Against COVID-19

The high mortality and morbidity from many respiratory diseases (30), including COVID-19, is attributed to sustained uncontrolled inflammatory infiltrates, antibody-dependent enhancement, and excessive cytokine production (cytokine storm) that lead eventually to lung tissue damage (31). The cytokine storm in COVID-19 patients is characterized by increased (IL)-1β and IL-6, IL-17, IFN-α, and INF-β, along with IL-37 and IL-38 (31). On the other hand, Ramadan diurnal intermittent fasting has a positive effect on the overall inflammatory status of the human body (24–27) and tends to decrease such pro-inflammatory cytokines, particularly IL-6, IL-1β (6, 7), and proinflammatory chemokines CXCL1, CXCL10, and CXCL12 (24–27), which might alleviate lung tissue damage. Ramadan intermittent fasting has a modulatory effect on macrophages and render them to produce low amounts of cytokines (32), previously proven to positively affect asthma patients (33). Of note is that fasting restored the balance of renin–angiotensin system (34, 35), which is crucial to reduce the effect of angiotensin II, pro-inflammatory cytokines, and fibrosis in the lung tissue (30). Taken together, these findings suggest that Ramadan diurnal intermittent fasting might have a favorable effect against COVID-19.

It should be noted that the beneficial effects of Ramadan intermittent fasting on immunity might be reduced by the sleep pattern practiced in the blessed month of Ramadan. Several studies showed that total sleep time significantly decreased by about 1 h in Ramadan nights, while daytime sleepiness increased (23, 36). Partial sleep deprivation is associated with increased susceptibility to viral infections (23, 37). It impairs the immune functions, decreases cytokine release, and reduces the infection-fighting antibodies and cells (38). Wilder-Smith et al. examined the effect of sleep deprivation on the immune markers of 52 healthy volunteers and showed its association with transiently impaired mitogen proliferation, decreased HLA-DR, upregulated CD14, and variations in CD4 and CD8 (39). A study by Bahijri et al. addressed the combined effect of Ramadan intermittent fasting and disturbed sleep to decrease the IgG level significantly in 23 healthy volunteers (40). Taken together, it is worth to state that the beneficial immunomodulatory effects of Ramadan intermittent fasting might be influenced by the disturbance of the sleep–wake cycle of fasting individuals.

## Periodic Fasting Preserves and Improves the Immunity

Recent studies showed that periodic fasting and time-restricted re-feeding would make the immune system stronger. Of note is that Muller et al. showed that the clinical use of periodic fasting reduces the symptoms of rheumatoid arthritis when followed by a vegetarian diet (41). Understanding the mechanistic link between nutrients and fasting benefits leads to the identification of fasting-mimicking diets (FMD) that achieve changes similar to those caused by fasting (41). Cheng et al. demonstrated the effect of periodic fasting in promoting a HSC-dependent regeneration of mice immune cells, leading to a rejuvenated immune phenotype and elevation of mesenchymal stem and progenitor cells (MSPC), even at a relatively old mice age (19, 41). Cycles of fasting and refeeding have been shown to modulate gut microbiota, ameliorate pathology in various mouse autoimmunity models, and promote T cell-dependent killing of cancer cells (42). Dang et al. showed that fasting enhances TRAIL-mediated liver natural killer cell activity against neoplastic cells through upregulation of HSP70 (17). In agreement with the anti-inflammatory effect of FMD in mice, Brandhorst et al. showed that the MSPC levels were transiently elevated during FMD in human subjects (41, 43). Interestingly, this intermittent fasting was practiced by Mohammed, the prophet of Islam, about 1,500 years ago.

## Concluding Remark

In light of the scientific evidence presented here, healthy adult Muslims will reap benefits from fasting and will improve body wellness and performance of the immune system, which will be a valuable asset in fighting against COVID-19 and is expected to expedite the healing process for patients with COVID-19.

## Data Availability Statement

The original contributions presented in the study are included in the article/Supplementary Material, further inquiries can be directed to the corresponding author/s.

## Author Contributions

HZ conceptualized the perspective idea, drafted the outline, and revised the text. TA and HA created the original perspective draft. All authors reviewed and commented on the subsequent drafts of the perspective and declare that the content has not been published elsewhere. All authors read and approved the final version.

## Conflict of Interest

The authors declare that the research was conducted in the absence of any commercial or financial relationships that could be construed as a potential conflict of interest.
