# Supplementary material for: Fasting Ramadan During COVID-19 Pandemic: Immunomodulatory Effect
Source: Front Nutr. 2020 Nov 5;7:557025. doi: 10.3389/fnut.2020.557025 (PMC7678565; doi:10.3389/fnut.2020.557025)
Supplement: Supplementary file 1 [file Table_1.DOCX]

Supplementary table 1. The immunomodulatory effect of different fasting patterns in experimental studies

| **First author, year**  **(reference number)** | **Experimental study Subjects** | **Fasting Type description** | **Immunomodulatory effect of fasting** |
| --- | --- | --- | --- |
| Cignarella, 2018 (9) | Female C57BL/6J mice & patients with relapsing-remitting multiple sclerosis (RRMS) | Alternate day fasting | Enrich gut microbiome, reduce IL-17A and INF-γ producing T-cells and increase regulatory T-cells in gut-associated lymphoid tissue, enhance antioxidative pathways. |
| Nagai , 2019 (11) | Male BALB/c mice | Alternate fasting/re-feeding for 24 to 36 hours | Induce apoptosis of germinal center and IgA+ B cells in Peyer’s patches, induce migration of naïve B cells to the bone marrow, decrease in CXCL 13 expression in Peyer’s patches |
| Jung , 2010 (13) | Mice | Diet restriction (protein-free diet) | Exhibit underdeveloped gut-associated lymphoid tissue with low amounts of IgA and systemic Th2 profile |
| Shushimita , 2014 (14) | Male C57Bl/6 mice | 30% diet restriction for 2 weeks and 3 days of water-only fasting | Induce maturation arrest of T-cells in thymus and depletion of mature T-cells from spleen and mesenteric lymph nodes while recruiting them in the bone marrow;  Induce significant reduction in pro-B, pre-B and immature-B cell population and significant increase  in recirculating mature B cells in the bone marrow; |
| Takakuwa , 2019 (15) | C57Bl/6 mice | Calorie restriction, short-term fasting with access to only water for 48 hours | Induces cell cycle arrest in immature hematopoietic cells and Increases naïve T cells in the bone marrow. |
| Collins , 2019 (16) | C57BL/6NTac mice | 50% diet restriction | Promote accumulation of memory T-cells in the bone marrow with energy conservation and enhanced function against infection; induce collapse of memory T-cells in secondary lymphoid tissue. |
| Dang , 2014 (17) | Female C57BL/6J mice | Free access to water with food deprivation for 1 or 3 days | Enhance TRAIL-mediated liver natural killer cell activity against neoplastic cells through upregulation of heat shock protein 70. |
| Cheng , 2014 (19) | C57BL/6J mice | Prolonged fasting for 48 hours during 6 cycles of chemotherapy | Reduces IGF-1/PKA activity to promote hematopoietic-stem-cell-based regeneration and self-renewal. |
| Contreras , 2018 (18) | C57BL/6 mice | Long term calorie restriction | Induce proliferation of CD8 naïve and central  memory cells most likely by IL-7 |
|  | 34 individuals | Long term calorie restriction | Decrease neutrophils, lymphocytes, and monocytes in peripheral blood. |
| Aksungar , 2007 (26) | 40 healthy individuals | Ramadan intermittent fasting for at least 16 h/day | Induce significant decrease of CRP and IL-6 |
| Faris , 2012 (24) | 151 healthy volunteers | Ramadan intermittent fasting for at least 21 days | Induce significant decrease of total leukocytes count, granulocytes, monocytes, and lymphocytes in peripheral blood, induce significant decrease of circulating IL-1β, IL-6, TNF-α |
| Almeneessier , 2019 (25) | 12 healthy male volunteers | Diurnal intermittent fasting for 1 week outside Ramadan, and 2 weeks of Ramadan fasting | Both induce significant decrease in the levels of cytokines IL-1β, IL-8 and IL-6. |
| Akrami Mohajeri , 2013 (27) | 58 healthy indviduals | Ramadan intermittent fasting for 14 hours | Induce decreased levels of pro-inflamatory CXC chemokines CXCL1, CXCL10 and CXCL12, induce significant reduction of IL-2, IL-8, and TNF-α |
| Sun , 2001 (32) | C57BL/6 Mice | Calorie restriction (CR) | Influence the expression of IL-12, IL-6, TLR2, TLR4, lipopolysaccharide receptor CD14 |
| Han , 2018 (33) | 18 mild-asthmatic subjects | Prolonged fasting for 24 hours initiated by an early morning fixed caloric meal with unrestricted water intake | Blunt the NLRP3 inflammasome and Th2 cell activation in steroid-naïve asthmatics, diminish airway epithelial cell cytokine production. |
| Brandhorst , 2015 (43) | C57BL/6 mice | Fasting mimics diet | Reduce cancer incidence and tissue inflammation, cause rejuvenation of immune cells |
